# Comparative sphingolipidomics of disease-causing trypanosomatids reveal unique lifecycle- and taxonomy-specific lipid chemistries

**DOI:** 10.1038/s41598-017-13931-x

**Published:** 2017-10-19

**Authors:** Xue Li Guan, Pascal Mäser

**Affiliations:** 1 Lee Kong Chian School of Medicine, Nanyang Technological University, Singapore, Singapore; 2 Swiss Tropical and Public Health Institute, Basel, Switzerland; 3 University of Basel, Basel, Switzerland

## Abstract

Trypanosomatids are parasitic protozoa which cause a spectrum of diseases, including trypanosomiasis and leishmaniasis, affecting millions of humans and animals worldwide. The surface of most protozoan parasites is heavily decorated with lipids and lipid-anchored molecules, forming protective barriers and acting as virulence factors during infection. Sphingolipids (SP) are major components of eukaryotic biomembranes, which play important roles in structural integrity, energy homeostasis and signaling. However, the precise chemical composition of SP in pathogens as well as their biochemical pathways and functions remain poorly characterized. Here, we present the first system-scale analyses of SP found in a panel of 7 trypanosomatids, including *Leishmania donovani*, *Trypanosoma brucei* and *Trypanosoma cruzi*. We characterized the structure of aminoethylphosphonate-containing ceramides, which are found exclusively in stercorarian *Trypanosoma*. Employing the sensitive and semi-quantitative sphingolipidomics approach that we developed, we report the detection of over 300 molecular species of SP, and identified unique metabolic signatures which serve as discriminants of the pathogens based on their taxonomy and lifecycle stages. The deep sphingolipidome presented here is an important biochemical and technological resource for future works to dissect SP metabolism and functions in these medically and agriculturally relevant systems.

## Introduction

Trypanosomatids are flagellated protozoan parasites, some of which have complex lifecycles alternating between an insect vector and a mammalian host. These include the Tritryp members *Trypanosoma brucei*, *Trypanosoma cruzi* and *Leishmania* species, causative agents for the devastating diseases African sleeping sickness, Chagas disease and leishmaniasis, respectively. Infection by trypanosomatids affect millions of people and countless animals worldwide in terms of public health and economy, with total human deaths of over 100 000 annually. Currently, there are no vaccines and the few available drugs display toxic side effects. Despite the breakthroughs ten years ago in the genomics analyses of the trypanosomatids and the identification of trypanosomatid-conserved and species-specific genes^1–4^, there remains a big translation gap between the genetic information, biological functions and clinical applications. Research on the pathways of parasites that do not have counterparts in the mammalian host may lead to identification of potential targets for the development of new therapeutics.

Sphingolipids (SP) are ubiquitous membrane components, which also serve as key mediators in energy homeostasis and signaling. SP are involved in diverse cellular processes, including differentiation and growth, and contribute to interactions between the pathogens and their hosts^5–7^. In *T. cruzi*, SP form structural components of lipid-anchored glycans and proteins, which serve as protective barriers and play critical roles in virulence and survival^8,9^. SP are synthesized *de novo* from the condensation of serine (and possibly other amino acids) and fatty acyl-CoA to form long chain bases (LCB)^10^, ultimately yielding ceramides, the precursors of more complex SP. These lipids undergo extensive remodeling during the life cycles of trypanosomatids^11–14^. Interestingly, perturbation of genes involved in the *de novo* step of SP biosynthesis in *L. major* and *T. brucei* leads to contrasting effects. Knockout of the serine palmitoyltransferase subunit SPT resulted in defects in differentiation but not viability in *L. major*, whereas inhibition of the enzyme led to lethality in the bloodstream form *T. brucei* ^15–17^. Additionally, rescue of the respective phenotypes was achieved by different metabolic intermediates, suggesting divergent metabolic requirements between parasite species^15,18^.

Intriguingly, SP biochemistry and structure is highly diverse between eukaryotic organisms. *Leishmania, T. brucei* and *T. cruzi* produce the fungal-like phosphosphingolipids (phosphoSP), inositolphosphoryl ceramides (IPC), which are absent in the mammalian hosts^19–23^. Besides IPC, it has been previously reported that the bloodstream form of *T. brucei* synthesizes phosphoethanolamine ceramides (EPC), the major phosphoSP found in *Drosophila melanogaster* ^13,24^ and a very minor component of mammalian SP^25^. Given the critical functional roles of protozoal SP and the presence of non-mammalian SP in trypanosomes, SP metabolism has been scrutinized as potential anti-parasitic drug targets^26–28^. However, there are currently no clinically available drugs that act through this pathway. The development is in part hampered by our limited knowledge on the detailed compositions as well as the molecular determinants of SP metabolism, regulation and functions in trypanosomes.

The identification and quantitation of the large number of individual SP molecules at high degree of structural accuracy are critical for the elucidation of SP metabolism and functions, with the increasing recognition that the fine compositional details of these lipids affect functions^29^. Here, we performed a systematic and in-depth characterization of trypanosomatid sphingolipidomes, based on liquid chromatography and tandem mass spectrometry (LC-MS/MS). We present the most comprehensive SP compositions of seven disease-causing trypanosomatids species including *T. brucei, T. cruzi* and *L. donovani*. We uncover the presence of phosphonate-containing SP in *T. cruzi* and *T. rangeli*, members of the stercorarian clade of *Trypanosoma*. We further demonstrate that the metabolic fingerprints can serve as taxonomic- and stage- specific discriminants of the pathogens. The detailed sphingolipidomes presented in this work are an important biochemical and technological resource for future works to dissect SP metabolism and functions in these medically and agriculturally relevant systems.

## Results

### Systematic qualitative analyses of trypanosomatid sphingolipids

**Figure 1:**
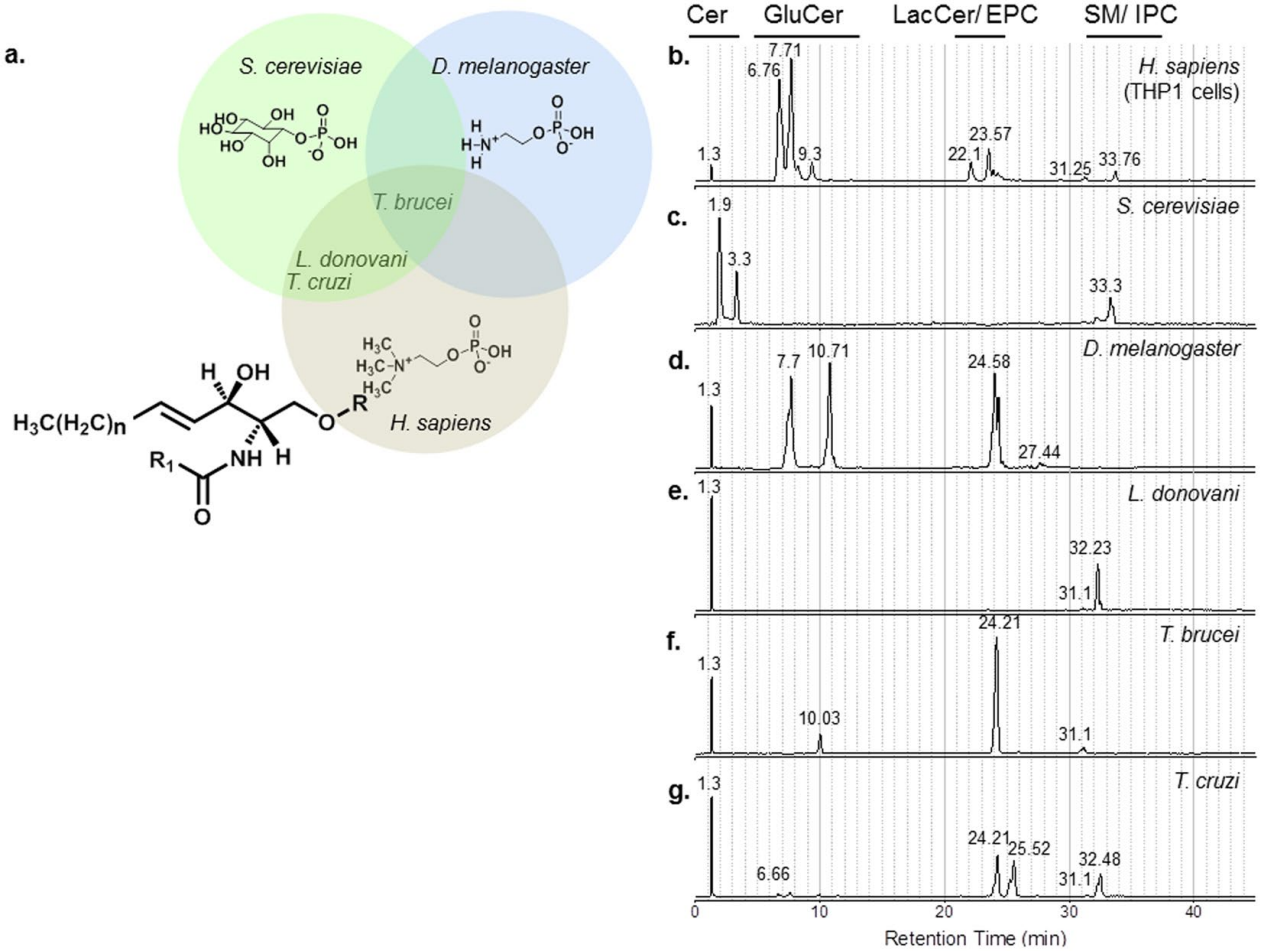
Distinct phosphosphingolipids (phospho-SPL) found in eukaryotic model organisms and trypanosomatids. (**a**) Structures of currently known phosphoSP found in human, *Saccharomyces cerevisiae*, *Drosophila melanogaster* and the trypanosomatids, *Leishmania donovani*, *T. brucei* and *T. cruzi*. Variations occur at the headgroup (**R**), long chain base (LCB) and fatty acyl (R_1_) chain. (**b–g**) Headgroup separation of SP in various organisms by liquid chromatography and precursor ion scans monitoring major LCB. (**b**) Chromatographic profile of SP found in human cell line, THP-1. (**c**) Chromatographic profile of SP found in *S. cerevisiae*. (**d**) Chromatographic profile of SP found in *D. melanogaster*. (**e**) Chromatographic profile of SP found in the trypanosomatid, *L. donovani*. (**f**) Chromatographic profile of SP found in the trypanosomatid, *T. brucei*. (**g**) Chromatographic profile of SP found in the trypanosomatid, *T. cruzi*. Abbreviations: Cer-ceramides, EPC-phosphoethanolamine ceramides, GluCer-glucosylceramides, IPC-inositolphosphoryl ceramides, LacCer-lactosylceramides, SM-sphingomyelins.

Previous reports of trypanosomatid SP revealed the presence of more than one phospho-headgroup^13,19,20,23^ (choline, inositol or ethanolamine), which are found in humans and the eukaryotic model organisms, *Saccharomyces cerevisiae* and *D. melanogaster*. We employed a combined normal-phase liquid chromatography and tandem mass spectrometry (MS/MS) method^24^ with modifications, to characterize the trypanosomatid SP. To validate our method, we capitalized on the natural diversity of phosphoSP found in humans^30^, yeast^31,32^ and the fruit fly^24^ and analyzed extracts derived from the human cell line, THP-1, and the two model organisms (Fig. 1b–d). The triple quadrupole instrument, operated in the precursor ion scan mode, selectively monitored ions containing a LCB to generate a qualitative fingerprint (Table S1). Clearly, chromatographic separation of SP with single headgroup modification was achieved in the following order: ceramides/dihydroceramides/phytoceramides (~1–2 mins, all 3 organisms), hexosylceramides (~6–11 mins, THP-1 and *D. melanogaster*), dihexosylceramides (~21.5–24 mins, THP-1), EPC (~23.5–25 mins, *D. melanogaster*), and IPC (~32–34 mins, *S. cerevisiae*) (Fig. 1b–d). Weak signals were observed at 33.76 mins in the human cell extract, arising from sphingomyelin (SM). It should be noted that sphingomyelin can be more sensitively detected by monitoring its phosphocholine headgroup (not shown)^33^.

We next applied this combinatorial LC-MS/MS approach to analyze the SP of the trypanosomatids, *Leishmania donovani* (axenic amastigotes), *T. brucei* (axenic bloodstream form) and *T. cruzi* (trypomastigotes). The major LCB of all three trypanosomatids (mammalian stage) is an 18-carbon sphingosine (d18:1) (Figure S1–S3). In contrast, insect stages of *Leishmania major* have been shown to predominantly produce d16:1 sphingosine by utilizing myristoyl CoA^21^, while Tanaka *et al*. detected the presence of d18:1 sphingosine-containing SP in amastigotes of *L. amazonensis* ^34^, suggesting stage- and/or species-dependent differences in SP chemistries. Based on retention times and collision-induced dissociation (CID), free ceramides and IPC were detected in *L. donovani* (Fig. 1e and Figure S1), whereas in the bloodstream form of *T. brucei*, free ceramides and EPC were observed (Fig. 1f and Figure S2). These findings were consistent with previously published reports^17,19^. It was noted that traces of glucosylceramides were detected in these parasite species.

Strikingly, for *T. cruzi* trypomastigotes, in addition to ceramides and IPC (Fig. 1g and Figure S3), we detected glucosylceramide, EPC and an additional peak eluting after EPC at approximately 25.5 mins (Fig. 1g). The relative complexity of the chromatographic profile of *T. cruzi* was surprising because only IPC and SM were previously described^35,36^ and the only sphingolipid synthase reported in *T. cruzi* to date is an IPC synthase^22^.

### Structural characterization and compositional confirmation of aminoethylphosphonate (AEPn)-containing SP in *T. cruzi*

**Figure 2:**
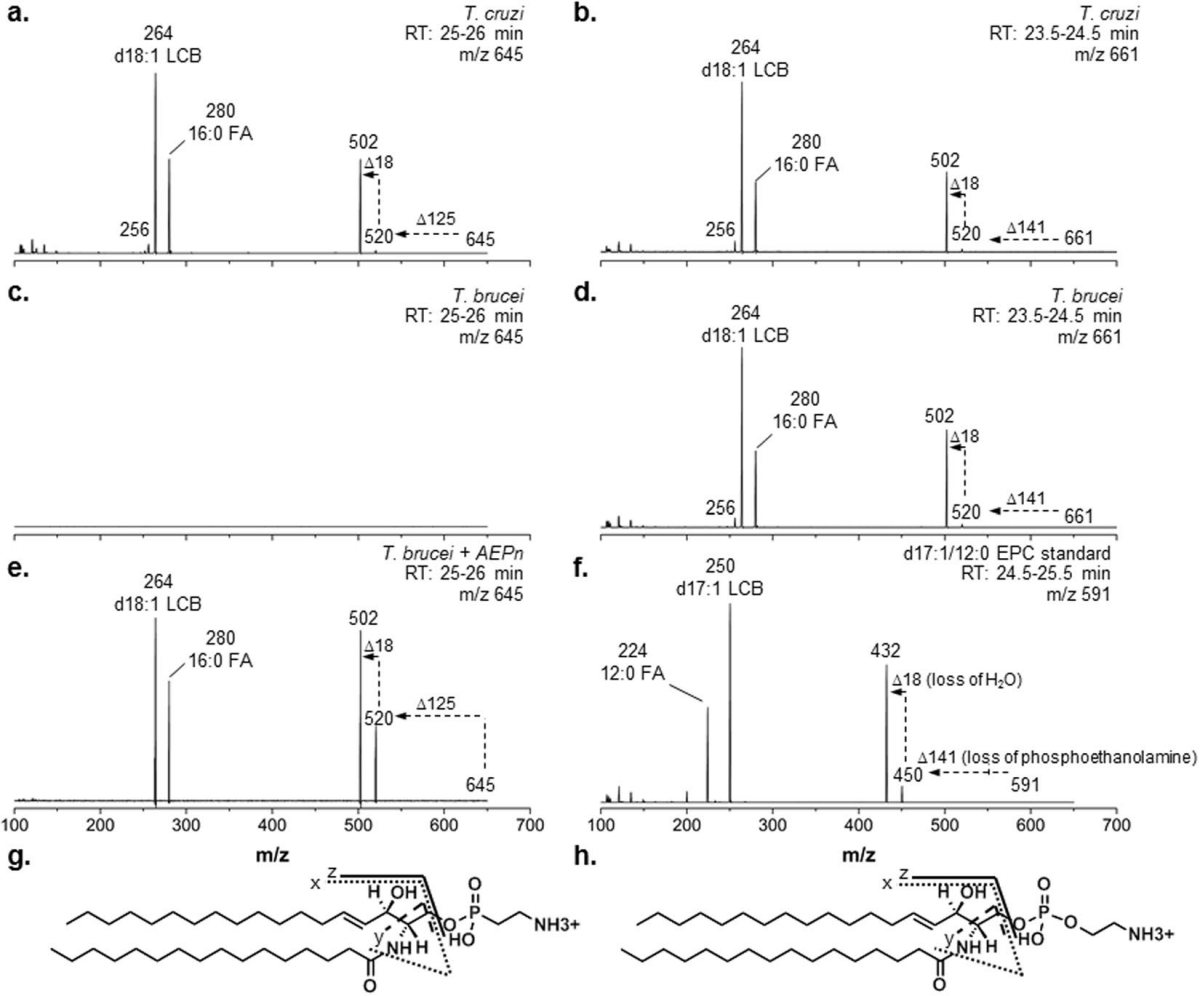
Characterization of aminoethylphosphonate (AEPn)-containing SP in *T. cruzi* by tandem mass spectrometry (MS/MS). (**a**) MS/MS of major ion found in *T. cruzi*, with m/z 645 and eluting between 25–26 minutes. Daughter ions include m/z 264, diagnostic of a d18:1 LCB, m/z 280 from a 16:0 fatty acyl chain, m/z 520 and m/z 502. (**b**) MS/MS of a d18:1/16:0 EPC, the major EPC found in *T. cruzi*, with m/z 661 and eluting between 24–25 minutes. (**c**) Absence of signals with m/z 645 from *T. brucei* at 25–26 minutes. (**d**) MS/MS of a d18:1/16:0 EPC, the major EPC found in *T. brucei*, with m/z 661 and eluting between 24–25 minutes,. (**e**) MS/MS of major ion, with m/z 645, found in *T. brucei* cultured in the presence of AEPn, eluting between 25–26 minutes. Fragmentation reveals several daughter ions including m/z 264, diagnostic of a d18:1 LCB, m/z 280 from a 16:0 fatty acyl chain, m/z 520 and m/z 502, which result from neutral loss of AEPn and subsequent loss of water. This confirms the *T. cruzi* lipid to be a d18:1/16:0 AEPnC. (**f**) MS/MS of a d17:1/12:0 EPC synthetic standard. Daughter ions include m/z 250, diagnostic of a d17:1 LCB, m/z 224 from a 12:0 fatty acyl chain, m/z 450 and 432, arising from neutral loss of phosphoethanolamine and subsequent loss of water. (**g**) Structure of d18:1/16:0 AEPn-containing ceramide (AEPnC). Sites of fragmentation by collision-induced dissociation are shown - x, y and z denotes fragments arising from the LCB, the fatty acyl moiety and ion from the neutral loss of the AEPn headgroup respectively. (**h**) Structure of d18:1/16:0 EPC. Sites of fragmentation by collision-induced dissociation are shown, where x, y and z denotes fragments arising from the LCB, the fatty acyl moiety and ion from the neutral loss of the phosphoethanolamine headgroup respectively.

We next sought to characterize in detail the *T. cruzi*-specific lipid. The major ion eluting at approximately 25.5 min had a mass-to-charge ratio (m/z) of 645.5 (Figure S3). CID generated a fragment ion of m/z 264 and 280, diagnostic of an 18-carbon sphingosine (d18:1 LCB) and a 16:0 fatty acyl (FA) chain. In addition, two ions with m/z 520 and 502 (Fig. 2a) were detected, which are derived from a neutral loss of 125 amu from the precursor (m/z 645), and subsequent loss of water. This pattern shared similarity to the fragmentation pattern of the major EPC species found in *T. cruzi*, with m/z 661.5 (Figure S3), which generated fragment ions with m/z 264, 280, 520, and 502 (Fig. 2b). These fragment ions arose from the d18:1 LCB, 16:0 FA, and neutral loss of the phosphoethanolamine headgroup (141 amu), with subsequent loss of water, respectively (Fig. 2h). The identity of EPC was verified with a synthetic d17:1/12:0 EPC standard (Fig. 2f). Overall, both the precursor ion (m/z 645) of the *T. cruzi*-specific lipid and its neutral loss mass (125 amu) differed from the major *T. cruzi* EPC precursor ion (m/z 661) and its neutral loss mass (m/z 141) by 16 amu (Fig. 2a,b). This mass difference of 16 amu suggests a potential difference of an oxygen atom in the headgroup structure, possibly indicative of an aminoethylphosphonate (AEPn) moiety.

Phosphonate-containing SP have been previously described in a few organisms^37,38^, including marine sponges^39^, the protozoan, *Tetrahymena pyrifomis* ^40^ and the bacterium, *Bacteriovorax stolpii* ^41^. The *T. cruzi* genome encodes the biosynthetic machinery, including the enzyme phosphoenolpyruvate mutase (PEP mutase), for producing aminoethylphosphonate (AEPn)^42^. In *T. cruzi*, AEPn can be incorporated into glycerophosphonolipids^43^ and as a modification in their GPI anchors^44^. However, to date, AEPn-containing SP has not been described in *T. cruzi*. We hypothesize that the ability of *T. cruzi* to make AEPn-containing SP is in part due to its biosynthetic capability to produce AEPn which can then be transferred to ceramide. This lipid is absent in *T. brucei* and *Leishmania* species since PEP mutase and consequently AEPn are absent^2^.

In order to confirm the identity of the *T. cruzi* lipid, a synthetic standard is required. Early works by Smith and O’Malley demonstrated that AEPn supplementation in the growth medium of *Tetrahymena* cells increased their phosphonolipid contents^40^. We speculated if *T. brucei*, were given AEPn, the cells may incorporate the substrate into endogenous ceramide and hence, serve as a biological producer for AEPnC. It should be noted that purified *T. brucei* ethanolamine-phosphate cytidylyltransferase (TbECT), which is involved in the Kennedy pathway, is unable to accept AEP as a substrate to form phosphonolipids^45^. However, in the context of whole cells, other enzymes, including sphingomyelin synthase-related proteins, as well as co-factors are present. We cultured the mammalian stage of *T. brucei* in media containing AEPn for two passages to adapt the parasite to the media prior to lipid analyses. From the spectrum, we observed weak signals for ions co-eluting with, and sharing the same m/z as, the *T. cruzi*-specific SP, which were absent in untreated *T. brucei* (Fig. 2c). The fragmentation of the major species (m/z 645) found in the AEPn-treated *T. brucei* (Fig. 2e) shared a similar pattern as the *T. cruzi* lipid. This confirmed the major ion eluting at ~25.5 mins in *T. cruzi* and in the AEPn-treated *T. brucei* to be a d18:1/16:0 aminoethylphosphonate ceramide (AEPnC) (Fig. 2g).

### SP profiles of *T. cruzi* and *T. brucei* showed distinct life-cycle dependence

**Figure 3:**
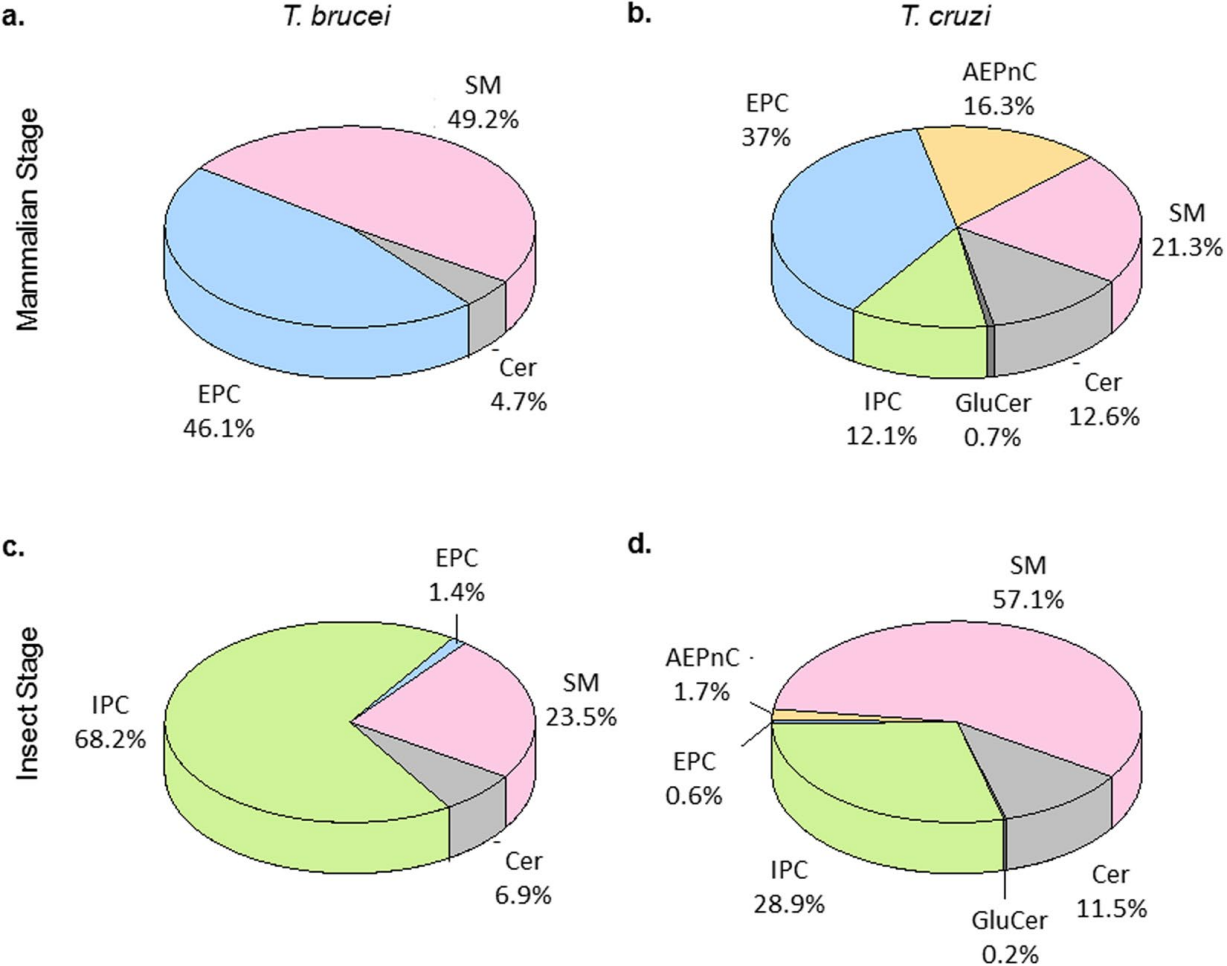
Extensive SP remodeling between mammalian and insect stages of *T. brucei* and *T. cruzi*. (**a**) Relative distribution of SP subclasses in the mammalian stage of *T. brucei* (bloodstream form). (**b**) Relative distribution of SP subclasses in the mammalian stage of *T. cruzi* (trypomastigotes). (**c**) Relative distribution of SP subclasses in insect stages of *T. brucei* (procyclic form). (**d**) Relative distribution of SP subclasses in the insect stage of *T. cruzi* (epimastigotes). AEPnC is present in the epimastigotes, albeit at lower levels. SP were measured using LC-MRM and the semi-quantitative data represent mol%, relative to the total SP measured.

It has been established in *T. brucei* that phosphoSP undergo dramatic remodeling during the lifecycle^13,14,46^. We next addressed whether the *T. cruzi*-specific SP signature is lifecycle stage-dependent. We optimized a semi-quantitative LC-MS/MS method, using multiple reactions monitoring (MRM), to selectively measure over 300 SP molecular species, spanning ceramides, glucosylceramides, EPC, AEPnC, IPC and SM. SP from trypomastigotes (mammalian stage) and epimastigotes (insect stage) of *T. cruzi* were analyzed. As a reference, comparative analysis was performed with the relatively well-characterized bloodstream form (mammalian) and procyclic form (insect) of *T. brucei* ^13^ (Fig. 3).

EPC and SM are the major phosphoSP in the *T. brucei* bloodstream form (Fig. 3a), while in the procyclic form of the parasite, IPC is relatively more abundant (Fig. 3c). Trace levels of EPC were detected in the procyclic form of *T. brucei*. The dramatic switches from EPC to IPC corroborated previous findings on the dynamic remodeling of *T. brucei* SP during its lifecycle^13,14^. Furthermore, it has been reported that four sphingolipid synthases (TbSLS1-4), which exhibit distinct substrate specificities, are responsible for the phosphoSP headgroup variations. TbSLS1 produces IPC and is uniquely responsible for synthesis of IPC in insect stage parasites, while TbSLS2 produces EPC. Both TbSLS3 and TbSLS4 are bifunctional SM-EPC synthases^47,48^.

In contrast, *T. cruzi* showed less exclusivity in terms of headgroup-specificity in its two extracellular lifecycle stages - all SP headgroups were detected in both stages, albeit in different proportions (Fig. 3b and d). AEPnC and EPC were abundant in trypomastigotes, while in the epimastigotes, the levels of these two subclasses were relatively low. Similar to *T. brucei*, the proportion of IPC increased in the insect stage of *T. cruzi*. Currently, only a single ortholog of sphingolipid synthase, TcSLS1, has been reported in *T. cruzi*. TcSLS1 is a dedicated inositol phosphorylceramide synthase^47^, and thus cannot solely account for the complex phosphoSP profile. Parasites including *Leishmania* and *Toxoplasma gondii* have been demonstrated to scavenge host SP^6,7,49^, including SM, and host SP as a source of *T. cruzi* SP remains to be further investigated. Nevertheless, our biochemical data clearly demonstrates that the two *Trypanosoma* species display distinct phosphoSP subclasses and different remodeling patterns between the mammalian and insect stages.

### AEPn-containing SP is present only in stercorarian *Trypanosoma*

We next questioned if AEPnC is specific to *T. cruzi*. Taxonomically, *T. brucei* and *T. cruzi* are distinguished by their clades - salivarian or stercorarian, respectively - based on the site of development within the insect vector. We expanded the analyses to related *Trypanosoma* species from the two clades amenable to laboratory cultivation. These include the salivarian *T. vivax*, *T. evansi*, *T. congolense*, *T. brucei rhodesiense*, *T. brucei brucei*, and the stercorarian *T. rangeli* and *T. cruzi*. All parasites were grown *in vitro*, except the animal pathogens, *T. congolense*, *T. vivax* and *T. evansi*, which were isolated from infected mice. To rule out any potential differences due to the method of production of salivarian *Trypanosoma*, we produced *T. brucei* subspecies from both axenic cultures and infected mice.

**Figure 4:**
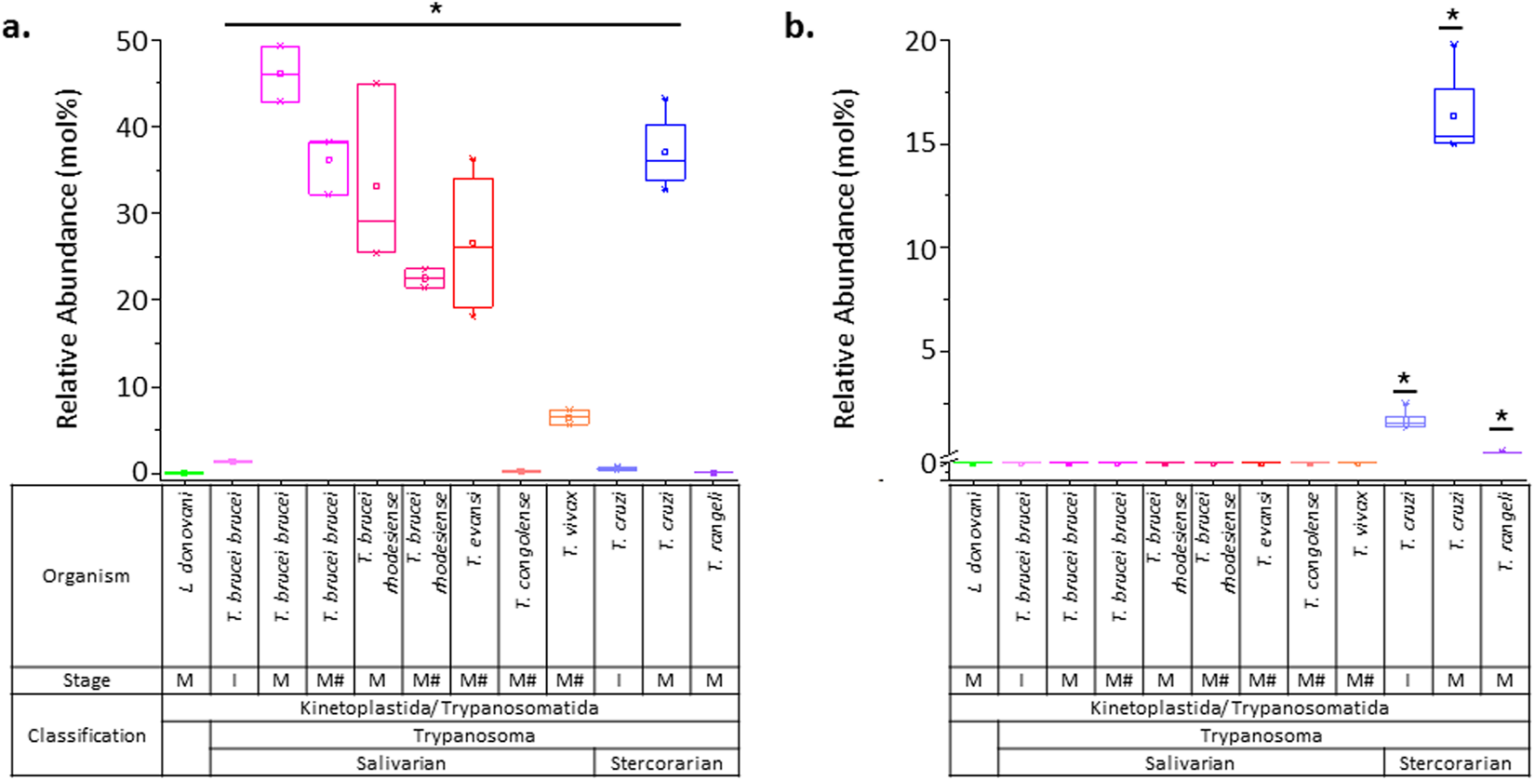
Presence of phosphoethanolamine- and AEPn-containing SP in human and animal disease-causing trypanosomatids. (**a**) Phosphoethanolamine ceramide (EPC) levels. (**b**) Aminoethylphosphonate ceramide (AEPnC) levels, which is specific to the stercorarian clade of *Trypanosoma*, *T. cruzi* and *T. rangeli*. Note: all parasite species were available in mammalian forms (abbreviated ‘m’), with the exception of *T. rangeli*, which was cultured in its insect form (abbreviated ‘i’). ^#^parasites isolated from mouse infection model. Error bars represent standard deviation. *p-value < 0.05, determined by Kruskal Wallis test.

Figure 4 shows the relative abundance of AEPnC across the panel of protozoan parasites. As a reference, we present the relative abundance of EPC since it differs structurally from AEPnC by one oxygen atom. In *L. donovani*, neither EPC nor AEPnC were present. While EPC was present in the mammalian stage of all *Trypanosoma* species and the insect stage of *T. cruzi* (Fig. 4a), AEPnC was detected only in the stercorarian *T. cruzi* and *T. rangeli* (Fig. 4b), albeit at relatively low levels in the epimastigotes. Together, these results confirm that amongst all the trypanosomatids analyzed, the presence of AEPnC was restricted to stercorarian *Trypanosoma*.

### Biochemical taxonomy of trypanosomatids based on SP

**Figure 5:**
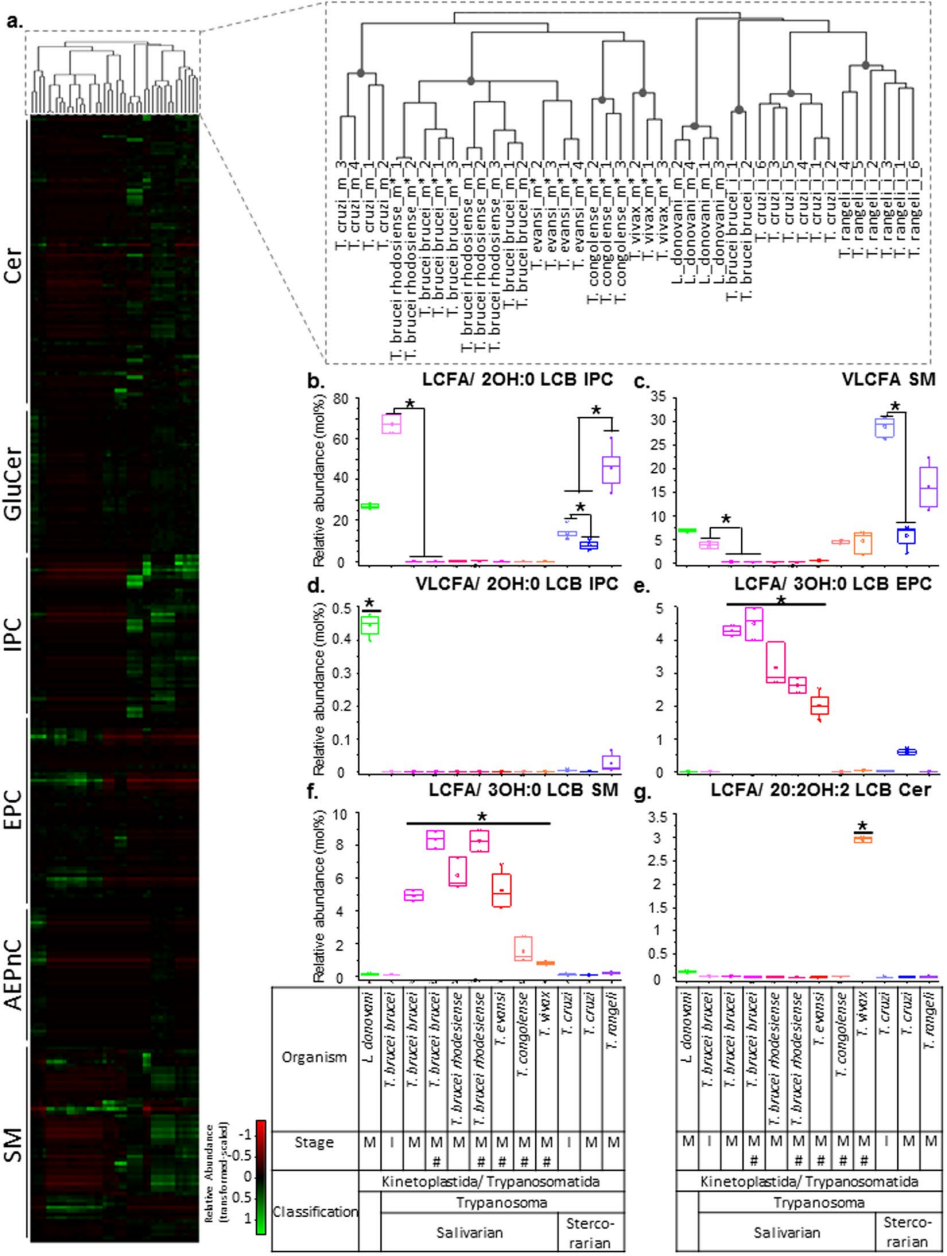
Taxonomic- and lifecycle stage-specific sphingolipid signatures of protozoan parasites. (**a**) Hierarchical clustering and heatmap representation of SP molecular species detected in the various protozoan parasites analyzed in this study. The data was scaled and transformed for comparison of the relative SP species levels across the different organisms and lifecycle stages. Green represents enrichment, and red represents de-enrichment of a lipid species across the various conditions. Enlarged cluster tree (top right) revealed clustering of parasites by organismal species and lifecycle stages, with grey dot representing branches where AU *p*-value > 95% (with multiscale bootstrap resampling, n = 10,000). The signature biochemical characteristics associated with specific organisms and/or lifecycle stages were analyzed and the relative abundance of the SP chemistries were represented using box plots. (**b**) LCFA/2OH:0 LCB-containing IPC are relatively higher in proportion in insect-stages of *Trypanosoma* species compared to their mammalian-stages. (**c**) VLCFA-containing SM are enriched in insect-stages of *T. cruzi* and *T. brucei* compared to their mammalian stages. (**d**) VLCFA/2OH:0 LCB-containing IPC are enriched in *L. donovani*, compared to other protozoan parasites. (**e**) LCFA/3OH:0 LCB-containing EPC are present and enriched in *T. brucei* (both subspecies) and *T. evansi*, but not the other salivarian *Trypanosoma*, *T. congolense* and *T. vivax*. (**f**) LCFA/3OH:0 LCB-containing SM are present in all salivarian *Trypanosoma* species, unlike LCFA/3OH:- LCB-containing EPC, which is more specific to the Trypanozoon subgenus. (**g**) Ceramide with LCFA and 20-carbon sphingadiene (20:2OH:2 LCB) are enriched in *T. vivax*. LCFA - long chain fatty acids; VLCFA - very long chain fatty acids; LCB chemistries are defined based on their chain length, degree of hydroxylation and unsaturation, for example 20:2OH:2 LCB denotes a LCB with 20 carbons, 2 hydroxyl groups, and 2 double bonds. Error bars represent standard deviation. *p-value < 0.05, determined by Kruskal Wallis test.

Another unexpected observation was the heterogeneity within the salivarian clade of *Trypanosoma* (Fig. 4a) based on the relative abundance of EPC. Notably, *T. congolense* and *T. vivax* had relatively lower levels of EPC in their total measured SP than *T. evansi* and *T. brucei* (both subspecies). Interestingly, *T. congolense* and *T. vivax* belong to the Nannomonas and Duttonella subgenus respectively, while *T. brucei* and *T. evansi* belong to the Trypanozoon subgenus^50^. We therefore examined how the protozoan species are separated based on the fine combinatorial chemistries of their SP molecular species (Table S2). Figure 5a shows the heatmap representation of the SP profiles across the panel of parasites and a tree generated from hierarchical clustering based on Euclidean distances.

The topology of the tree reveals several distinct clusters (Fig. 5a) grouping the various organisms according to species and lifecycle stages, with eight branches where the approximately unbiased (AU) *p*-value was greater than 95%. We next examined the SP lipid chemistries that were enriched in specific organisms of lifecycle stage. SP structural variations can occur at the headgroup as well as the FA chain length (long chain fatty acid, LCFA (12–20 carbons) vs very long chain fatty acid, VLCFA (21–26 carbons)), LCB length (14–20 carbons), and degree of hydroxylation (2 or 3 hydroyxl (OH) groups) and unsaturation (0, 1 or 2 double bonds), which can be differentiated with the sensitive and high resolution LC-MS/MS method.

The insect (i) forms of *Trypanosoma* clustered separately from the mammalian (m) forms of *Trypanosoma*. The insect forms of the three *Trypanosoma* species, *T. brucei brucei*, *T. cruzi* and *T. rangeli*, exhibited higher relative amounts of IPC with LCFA and dihydrosphingosines (2OH:0), compared to the mammalian stage (Fig. 5b). This difference was less striking in *T. cruzi* (1.74-fold, c.f. *T. brucei* >100-fold). The proportion of IPC with LCFA and dihydrosphingosine (2OH:0) in *T. rangeli* was 3.35-fold higher than in *T. cruzi*, and hence differentiating the two organisms. A more distinct separation of the insect and mammalian forms of *T. cruzi* was observed based on their relative proportion of SM with VLCFA (Fig. 5c). Both these IPC and SM chemistries were, however, not unique as they were shared with *L. donovani* amastigotes, which clustered with the insect stages of the three *Trypanosoma* species. *L. donovani* can be discriminated from the latter as it had relatively higher levels of IPC containing dihydrosphingosine (2OH:0) and VLCFA (Fig. 5d). It should be noted that *Leishmania* do not synthesize SM, but have the ability to scavenge host SP^6^. However, our current analyses will not be able to discriminate between *de novo* synthesized and host- or media-derived lipids, and the source of SM detected in all the analyzed protozoan parasites requires further investigation.

Within the mammalian forms of *Trypanosoma*, *T. cruzi* separated from the salivarian species due to the presence of AEPnC (Fig. 4b). With respect to the salivarian clade, *T. brucei* subspecies clustered with *T. evansi*, while *T. congolense* and *T. vivax* formed separate branches, correlating with separation by their subgenus. While we noted differences in the relative abundance of the EPC present in the salivarian *Trypanosoma*, we found that species of the subgenus Trypanozoon were enriched in EPC with LCFA and phytosphingosine (3OH:0) (Fig. 5e). Examination of SM with LCFA and phytosphingosine (3OH:0), which was present in all the salivarian *Trypanosoma* species (Fig. 5f), suggested that the enrichment of EPC chemistry in *T. brucei* subspecies and *T. evansi* is specific. *T. vivax* formed a separate cluster from all the other salivarian *Trypanosoma*, in part due to the enrichment of ceramides species containing LCFA and 20-carbon sphingadiene (20:2OH:2) (Fig. 5g).

Overall, our data demonstrate the complexity of the sphingolipidomes and correlation of the fine metabolite signature with the lifecycle stages and phylogeny of the *Trypanosoma* species.

## Discussion

In this work, we documented in a systematic fashion the relative abundance of more than 300 SP molecular species, representing the most comprehensive sphingolipidome analyses of seven trypanosomatid species. This is the first systems-scale level characterization and semi-quantification of the SP of a spectrum of medically and agriculturally important eukaryotic parasites. While the method is extremely sensitive, permitting the identification and detection of previously known as well as unknown SP, it remains semi-quantitative (i.e. does not reflect absolute quantities) due to a lack of pertinent internal standards for the phosphoSP, IPC and AEPnC. Nevertheless, we clearly demonstrated that the high resolution combinatorial chemistries of the SP reported in this study serve as a discriminant of the biology of the trypanosomatids, following the lifecycle stages and phylogeny of the parasites analyzed. It should be noted that our analysis is unable to discriminate between host-derived vs *de novo* synthesized lipids, which can be potentially overcome using metabolic labeling strategies^7^. Another limitation of this study is the coverage, in terms of the panel of organisms studied, as well as the SP chemistries, which is non-exhaustive. This work focuses on single headgroup modifications of ceramides but does not include complex glycosphingolipids (with more than one sugar), or the signaling and soluble molecules, free LCB, their phosphorylated products and ceramide-1-phosphate. The latter less hydrophobic and naturally low abundant SP require alternative isolation and analytical methods, including reverse phase chromatography^51^.

This study also reported for the first time the structural characterization of a phosphonate-containing SP, AEPnC, in *T. cruzi*. Confirmation of the AEPnC structure was achieved through ‘*in vitro* synthesis’ of AEPnC by cultivating bloodstream form of *T. brucei* with AEPn. Interestingly, weak but detectable signal of AEPnC was observed using our sensitive LC-MS/MS method, highlighting the specificity of the *T. brucei* enzymes^52^ which potentially accounts for the poor incorporation of AEP into AEPnC. Nonetheless, we demonstrated that AEPnC is present in the stercorarian trypanosomes, *T. cruzi* and *T. rangeli*, but not in members of the salivarian trypanosomes or *L. donovani*. The presence of AEPnC in *T. cruzi* is consistent with the presence of phosphoenolpyruvate mutase in its genome^3,42^. The natural existence of phosphonates has been discovered only 50–60 years ago mainly in marine life forms and prokaryotes^53^ Phosphonates are an interesting class of compounds with a characteristic direct carbon-phosphorus (C-P) bond, which is highly stable compared to the labile carbon-oxygen-phosphorus linkage found in phosphate^54^. The C-P bond confers structural rigidity and resistance to chemical and enzymatic breakdown. Little is known about phosphonate functions in eukaryotes, particularly for AEPC. Open questions include whether this lipid is essential for *T. cruzi* and if the phosphonate chemistry affects membrane properties and cellular functions in the parasite. Given the absence of AEPnC, and more specifically PEP mutase in the human host, unraveling new insights into the biosynthesis and functions of this class of molecules are potentially relevant for possible intervention and diagnosis of Chagas disease.

In addition to the discovery of AEPnC in *T. cruzi*, the structural specificity of the various phospho(no)SP in the different trypanosomatids we observed is intriguing. While eukaryotes share common phospho-headgroup moieties (choline, inositol, ethanolamine, serine, glycerol) in their glycerophospholipids, our systematic analyses showed that this does not apply to SP, which exhibit organism-dependent variations. The transfer of the phospho-headgroups is mediated by sphingomyelin synthase (SMS) and SMS-related proteins (SMSr)^55^, and in the case of yeast, inositol phosphoryltransferase^56^. The sphingolipid synthase from *Leishmania* has been cloned and functionally confirmed to be an IPC synthase^47,57^, whereas the *T. brucei* genome encodes 4 SMSr proteins (TbSLS1-4) which transfer phosphocholine, phosphoethanolamine and phosphoinositol to ceramide. The substrate preference of these enzymes is dependent on their amino acid sequence^52^. Counterintuitively, although we found in this study that *T. cruzi* SP are decorated with the most numbers of phospho(no)-headgroups, *T. cruzi* only has one SMS-like gene annotated in its genome^3^, which has been previously characterized to be an IPC synthase^22,47^.While we do not rule out possible remodeling and acquisition of SP from the host or environment, which is the case for *Leishmania*’s ability to acquire SM^7^, this does not account for the inositol and phosphonate-containing SP, which are specific to the parasite and absent in the mammalian host or culture media. Mammalian cells also produce small quantities of EPC^25^ but due to the low abundance of this lipid in the host, it is unlikely to be a major and direct contributor of parasite EPC. Our biochemical data overall suggest that the *T. cruzi* genome may encode other enzymes involved in SP biosynthesis.

**Figure 6:**
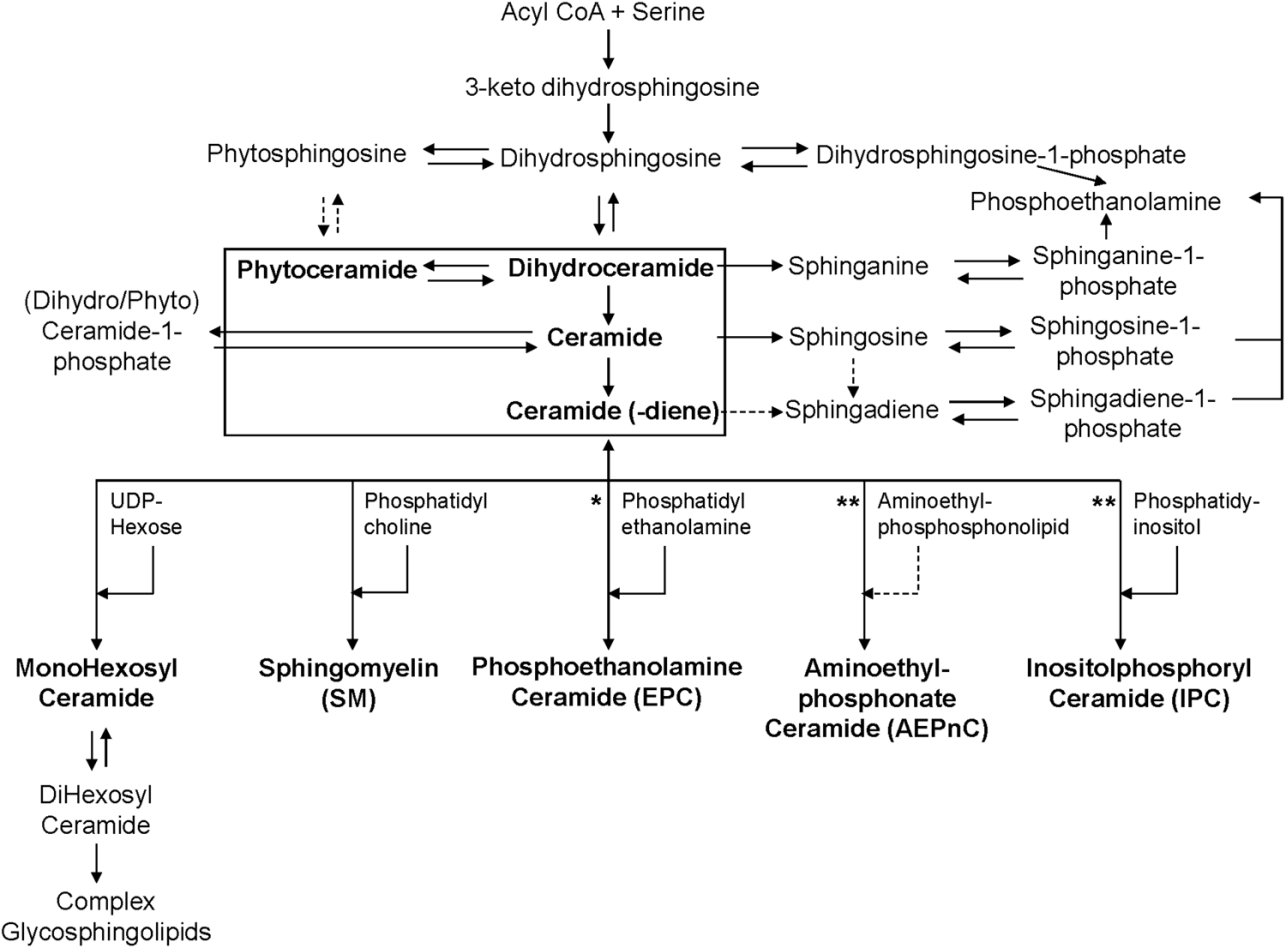
Proposed SP pathway of eukaryotic protozoan parasites based on the detailed sphingolipidome analysis (bold) performed in this study. The pathway takes into consideration the variations of the headgroup, FA length and degree of unsaturation as well as the LCB chain length and degree of unsaturation and hydroxylation. The enzymes involved in most steps remain to be identified and characterized. Some of the pathways are found in human (host) but a few (denoted by **) are unique in some parasite species, including the synthesis of AEPnC and IPC. *EPC is found in low abundance in mammalian cells, but highly enriched in several parasites.

Beside the structural diversity of SP headgroups, the LCB and FA chemistries are also distinct between the various parasite species and lifecycle stages. For instance, *T. vixax* was enriched in sphingadiene-containing ceramides as compared to all the other trypanosomatids analyzed. Evidently, it is the overall combination of the fine chemistries of the polar headgroups and hydrophobic tails, achieved with the deep sphingolipidome analyses, which allowed the discrimination of the various parasites. Based on the metabolites detected in this work, taking into consideration the headgroup and hydrophobic tail diversity, we propose the general SP pathway for the protozoan parasites analyzed (Fig. 6). While the enzymes involved in the various steps of the pathway remain to be annotated, the analytical tool developed and biochemical information generated will serve as a reference for future studies of SP metabolism and functions in trypanosomatids.

The striking association of the SP profiles with phylogeny and lifecycle stages of the parasites brings with it the question of the evolutionary origin of SP diversity. While this would require a broader spectrum of organisms to be analyzed, which is beyond the scope of this study, we postulate that the observed metabolite differences are important for functions, including adaptation to the environment (oxygen, pH, temperature) that the parasites are exposed to. Indeed, based on works on *S. cerevisiae*, cells respond to heat stress by adjusting their SP levels^58^. Our data on the lifecycle-dependent changes in SP profiles, particularly headgroup modification and LCB saturation, suggest possible adaptation to the hosts and environment. For instance, the body temperature of the vector is approximately 25 °C whereas for the mammalian host, it is approximately 37 °C. We also observed the contrasting differences in the SP switches between *T. brucei* and *T. cruzi* lifecycle stages, suggesting differential adaptation in their distinct hosts. It should be noted that the *T. cruzi* trypomastigotes do not replicate extracellular but require a host cell whereas *T. brucei* replicate freely in the blood. An exacting analysis of the ecology of the various parasites and their lipid composition could lead to a better understanding of SP evolution and the functional implications of the structural divergence.

In summary, the systematic and in-depth sphingolipidomics approach, in combination with classical parasitology, unveiled the fine metabolic signatures of a group of medically and agriculturally important eukaryotic parasites. The system-scale bioanalytical platform provides a technical resource for parasite SP analyses. Additionally, the comprehensive biochemical dataset generated can be further mined for identification and characterization of pathogen-specific pathways. Overall, this study will facilitate future investigations for a better understanding of the metabolism and functions of SP in trypanosomatids. Unique metabolite profiles and parasite-specific pathways in SP remodeling can eventually be evaluated as potential biomarkers and/or drug targets.

## Methods

### Analytical reagents

All organic solvents and additives for LC-MS/MS analyses were of LC or LC-MS grade and were obtained from Sigma-Aldrich. Other chemicals were of analytical grades.

### Cell and Parasite Cultivation

Parasites used in this study include *Trypanosoma brucei brucei* (STIB345), *Trypanosoma brucei rhodesiense* (STIB 900), *Trypanosoma cruzi* (Y-strain Osuna), *Trypanosoma evansi* (STIB806K), *Trypanosoma congolense* (STIB736/1180), *Trypanosoma rangeli* (S. Agustin), *Trypanosoma vivax* STIB719/ILRAD560), and *Leishmania donovani* (MHOM-ET67/L82).

Bloodstream forms of *T. brucei brucei* were maintained in HMI-9 medium with 10% horse serum. Procyclic forms of *T. brucei brucei* were maintained in SDM-79 medium with 10% fetal calf serum (FCS). Trypomastigotes of *T. cruzi* were obtained from the supernatant of infected microtus embryonic fibroblasts (MiEF) cultured in RPMI containing 10% FCS. The *T*. *cruzi* trypomastigotes released into the supernatants of infected MiEF cells were pelleted by centrifugation (1000 *g*, 10 min) to remove intact MiEF cells and collected from the supernatant after swimming up from the pellet 4 hours post-incubation at 37 °C, 5% CO_2_^59^. Epimastigote forms of *T. cruzi* were maintained in liver infusion tryptose medium containing 10% FCS and 2 µg/mL hemin. Insect stage of *T. rangeli* was maintained in SM medium containing 20% FCS and 2 µg/mL hemin_._ *L. donovani* axenic amastigotes were maintained in SM media (pH 5.4) containing 10% FCS. All axenic parasite cultures were grown in incubators maintained at 95% humidity and 5% CO_2_, with insect forms grown at 27 °C, and mammalian forms grown at 37 °C. All samples were harvested at the exponential phase of growth.

Trypanosomes isolated from blood of infected mice were purified using diethylaminoethanol-anion exchange chromatography. All samples were harvested by centrifugation, followed by three washes with phosphate-buffered saline (PBS) to reduce contamination from culture media. The samples were then snap frozen in liquid nitrogen and stored in −80 °C for subsequent lipid extraction.

For AEPn-treatment of bloodstream form of *T. brucei*, 1 mM of AEPn was added to the culture media and *T. brucei* was cultivated for 2 passages in AEPn-containing media before harvesting for lipid analyses.

### Lipid standards

d18:1/12:0 SM, d18:0/12:0 SM, d17:1/12:0 EPC and d18:1/8:0 Glucosylceramide, were obtained from Avanti Polar Lipids (Alabaster, AL, USA). Dioctanoyl (diC8:0) phosphatidylinositol (PI) was purchased from Echelon Biosciences, Inc. (Salt Lake City, UT, USA). d18:1/19:0 ceramide was obtained from Matreya LLC (Pennsylvania, PA, USA). Amino-ethyl phosphonic acid (AEPn) was obtained from Sigma-Aldrich.

### Lipid extraction

Samples were resuspended in 100 µL of PBS and lipids were extracted using a modified Bligh and Dyer’s method^60^. Briefly, 600 µL of chloroform:methanol (1:2, v/v) was added and the mixture was vortexed for one minute. The samples were placed on a thermomixer and shaken at 1000 rpm for 2 hours at 4 °C. 200 µL of chloroform and 250 µL of water were added to the mixture, which was vortexed for one minute. The phases were separated by centrifugation, and the lower organic phase was collected. The aqueous phase was re-extracted and the two organic extracts were pooled and concentrated by drying under vacuum using a Centrivap (Labconco Corporation, Kansas City, MO). The dried lipid film was re-suspended in 100 µL of chloroform-methanol (1:1, v/v) and used for LC-MS and LC-MS/MS analyses.

### Analysis of SP using liquid chromatography and mass spectrometry

A high performance liquid chromatography system (Agilent 1260) coupled with a triple quadrupole mass spectrometer (Agilent 6460) operated in positive electrospray ionization mode was used for characterization and semi-quantification of SP. SP were separated using normal phase chromatography^24^. The MS conditions were as follows: Gas temperature: 300 °C, Gas Flow: 10 L/min, Sheath Gas Temperature 350 °C, Sheath Gas Flow: 8 L/min and Capillary Voltage: 4000 V.

### Profiling and characterization of SP by LC-MS/MS

SP were resolved using precursor scans of dehydrated LCBs^24,33^ (Table S1). Further characterization and confirmation of the SP molecular details were performed using product ion scans, with collision energy ranging from 25 V to 40 V.

### Targeted semi-quantification of SP by LC- multiple reactions monitoring (MRM)

Based on the product and precursor ion analyses of headgroups and LCBs, a list of multiple reactions monitoring (MRM) transitions was generated to follow SP molecular species. Ceramides were measured using the parent > LCB fragment transitions, and all other SP were measured using the parent > headgroup fragment transitions. Each individual ion dissociation pathway was optimized with regard to collision energy and fragmentor voltage to minimize variations in relative ion abundance due to differences in dissociations^33,61^ associated with structural variations of the lipids. Lipids were separated using liquid chromatography with the above conditions.

For semi-quantitative analyses by MRM, prior to analyses of the samples, a cocktail of internal standards was spiked into each sample, adjusted based on the total ion counts. Final dilution of the samples were adjusted to 50 µL per 100 000 parasites, and 7 µL of samples were injected and separated by normal phase chromatography. For all SP subclasses analyzed, standards were available, except for AEPnC and IPC, which were normalized to EPC and PI standards respectively.

Analysis was carried out in at least biological triplicates (n = 3 to 7) for each organism, except for *T. brucei brucei* axenic cultures and *T. brucei* rhodesiense isolated from infected mice (n = 2).

### Data analyses

Data processing, including peak smoothing and integration of areas under the curve (AUCs) for each ions measured, was performed using the MassHunter Quantification Software (Agilent). As a control for background, extraction was carried out using PBS only. Relative abundance of each SP species and subclass were obtained by normalizing to the respective internal standards and further calculations using the following formulae:

Mol% of SP species 1 as a function of total SP measured = SPL1 (mol)/Sum of all SP measured (mol)

Mol% of SP subclass as a function of total SP measured = Sum of SP species of a class (e.g. all ceramide species, in mol)/Sum of all SP measured (mol)

Comparisons of the means of lipids were performed between conditions (between parasites, between strains, insect vs mammalian stages, axenic culture vs *in vivo* isolated parasites). Data are expressed as means with standard deviations as indicated by the error bars. Non-parametric Kruskal-Wallis test was used to determine the significance of difference in SP levels between conditions.

### Hierarchical clustering of lipid species

The MRM data from all samples (parasite species, lifecycle stages) were treated independently. Prior to hierarchical clustering, transformation and rescaling of the data were computed, applying arcsin and pareto scaling respectively^62^. Subsequently, clustering analysis was performed using Euclidean distances with average-linkage implemented in the open-source clustering software Cluster 3.0^63^. The dendrogram and heatmap of the computed data was visualized using the open-source software Java TreeView^64^. Approximately unbiased (AU) p-values were estimated using multiscale bootstrap resampling (n = 10,000) by the pvclust package implemented in R^65^ (R Development Core Team, 2010). Average of the relative abundance of lipid groups that are enriched in a given ‘organism(s)’ and/or ‘lifecycle stage’ clusters as well as the standard deviations were plotted in OriginPro. Non-parametric Kruskal-Wallis test was used to determine the significance of difference between specified conditions.

### Ethical Statement

All protocols and procedures involving animals were reviewed and approved by the local veterinary authorities of the Canton Basel-Stadt, Switzerland (permit No. 2813). All experiments were performed in accordance with relevant guidelines and regulations.

### Data availability

Supplementary Table 1 has also been made available at https://sites.google.com/site/lipidsinsystemshealthlab/resources-1/parasite-sphingolipidome.

## Electronic supplementary material

Supplementary Figures and Legends

Supplementary Table S2
