# Supplementary material for: Comparative sphingolipidomics of disease-causing trypanosomatids reveal unique lifecycle- and taxonomy-specific lipid chemistries
Source: Sci Rep. 2017 Oct 19;7:13617. doi: 10.1038/s41598-017-13931-x (PMC5648825; doi:10.1038/s41598-017-13931-x)
Supplement: Supplementary file 1 — Supplementary Figures and Legends [file 41598_2017_13931_MOESM1_ESM.pdf]

Comparative sphingolipidomics of disease-causing trypanosomatids reveal unique lifecycle- and taxonomy-specific lipid chemistries.

Guan and Maeser.

Supplementary Materials

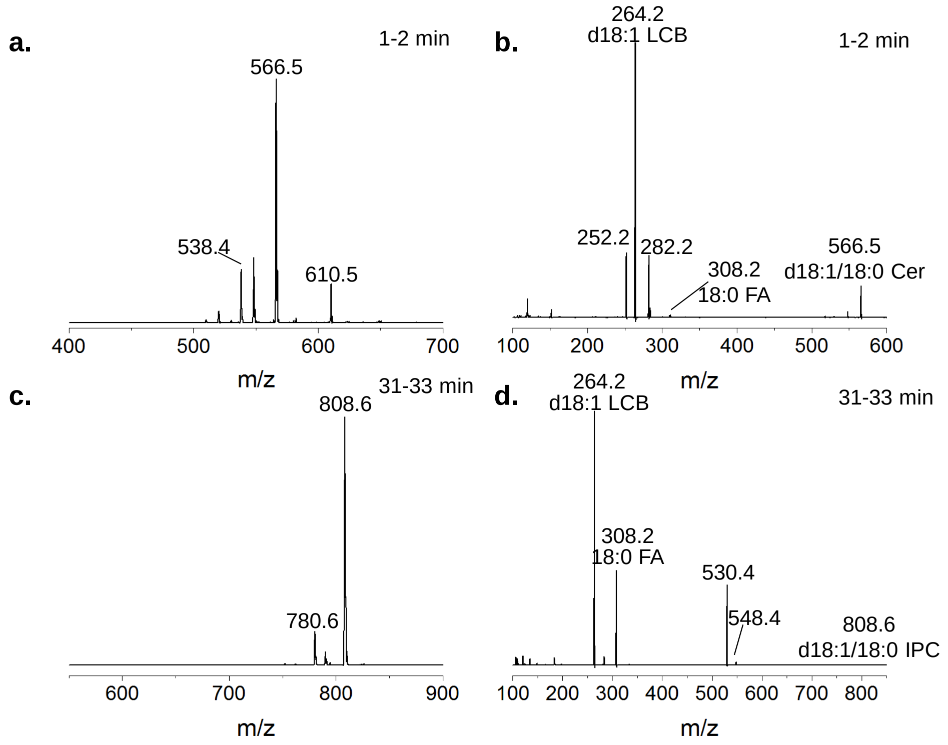

Guan and Maeser. Figure S1

Figure S1. Characterization of *L. donovani* SP. (a) Extracted spectrum within the 1-2 minutes retention time range from the LC-precursor ion scan, showing the d18:1 LCB-containing ceramides profile. (b) MS/MS spectrum of the major ceramide species, d18:1/18:0 Cer, with m/z 566. (c) Extracted spectrum within the 31-33 minutes retention time range from the LC-precursor ion scan, showing the d18:1 LCB-containing IPC profile. (d) MS/MS spectrum of the major IPC species, d18:1/18:0 IPC, with m/z 808. The retention time of IPC is earlier than the yeast counterpart as yeast contains SPL with higher degree of hydroxylation, which result in longer retention.

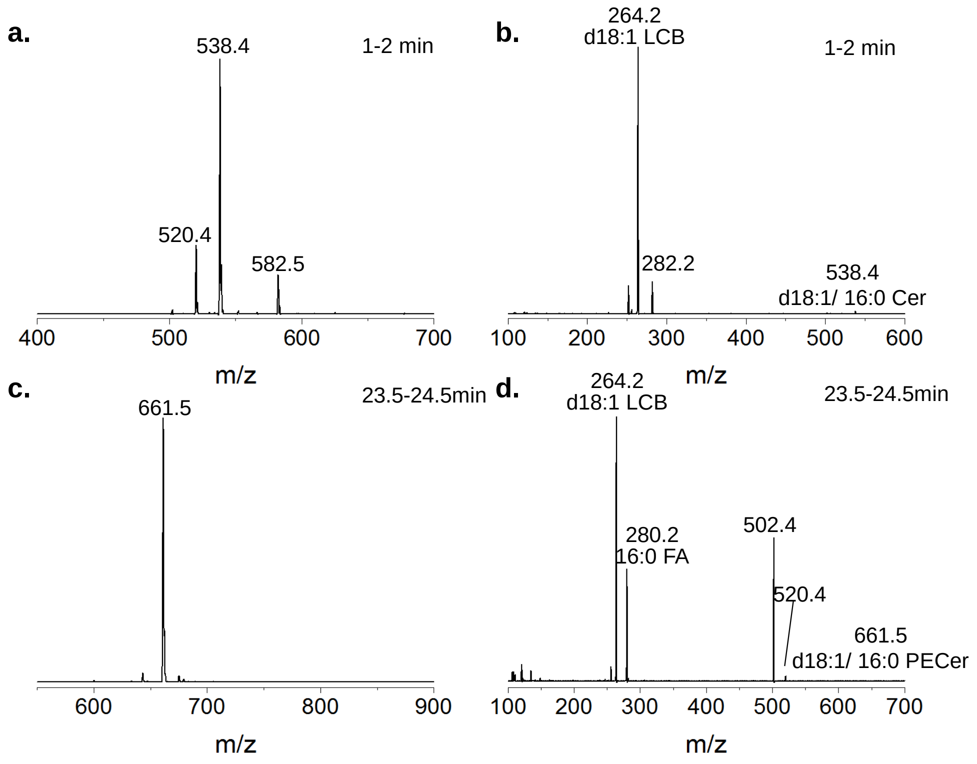

Guan and Maeser. Figure S2

Figure S2. Characterization of *T. brucei* SP. (a) Extracted spectrum within the 1-2 minutes retention time range from the LC-precursor ion scan, showing the d18:1 LCB-containing ceramides profile. (b) MS/MS spectrum of the major ceramide species, d18:1/16:0 Cer, with m/z 538. (c) Extracted spectrum within the 23.5-24.5 minutes retention time range from the LC-precursor ion scan, showing the d18:1 LCB-containing EPC profile. (d) MS/MS spectrum of the major EPC species, d18:1/16:0 EPC, with m/z 661.

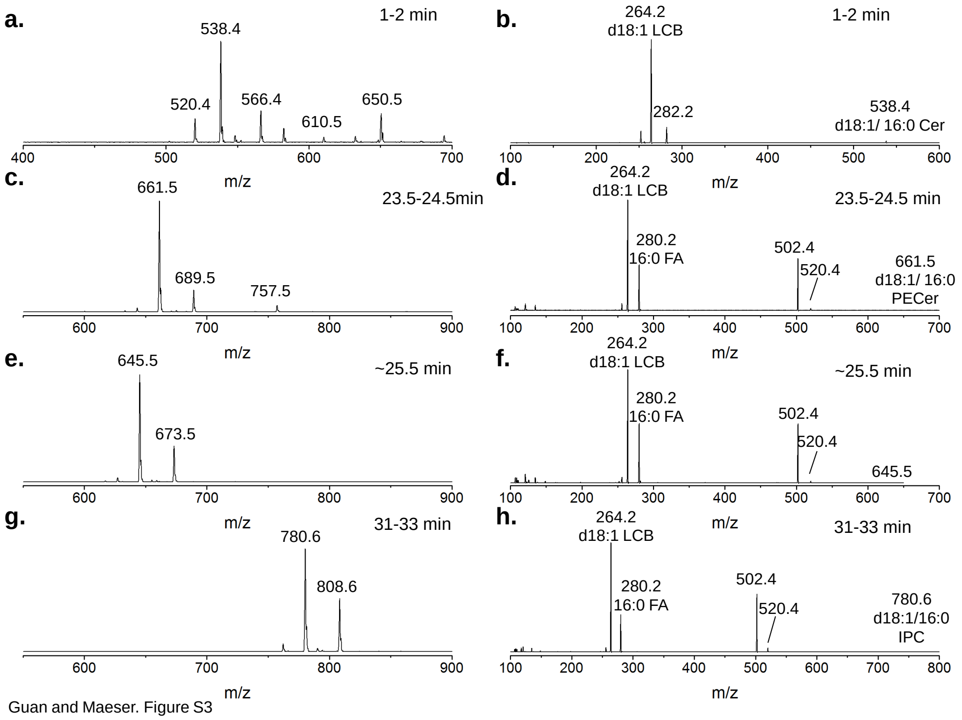

Figure S3. Characterization of *T. cruzi* SP. (a) Extracted spectrum within the 1-2 minutes retention time range from the LC-precursor ion scan, showing the d18:1 LCB-containing ceramides profile. (b) MS/MS spectrum of the major ceramide species, d18:1/16:0 Cer, with m/z 538. (c) Extracted spectrum within the 23.5-24.5 minutes retention time range from the LC-precursor ion scan, showing the d18:1 LCB-containing EPC profile. (d) MS/MS spectrum of the major EPC species, d18:1/16:0 EPC, with m/z 661. (e) Extracted spectrum at retention time range of ~25.5 min, from the LC-precursor ion scan. (f) MS/MS spectrum of the major ion eluting at ~25.5 min, with m/z 645. Detailed description of characterization of this *T. cruzi*-specific peak is described in text. (g) Extracted spectrum within the 31-33 minutes retention time range from the LC-precursor ion scan, showing the d18:1 LCB-containing IPC profile. (h) MS/MS spectrum of the major IPC species, d18:1/16:0 IPC, with m/z 780. The retention time of IPC is earlier than the yeast counterpart as yeast contains SPL with higher degree of hydroxylation, which result in longer retention.

Table S1. List of *m/z* ([M+H]+) for long chain bases (LCB) ranging from 14 to 20 carbons in length, with different degrees of saturation and hydroxylation.

**Long chain base**	**Alternative nomenclature (# of carbon:# of hydroxylation: # of double bond)**	**Product ion [M+H]+**
d14:0	14:2OH:0	210.2
d14:1	14:2OH:1	208.2
d14:2	14:2OH:2	206.2
t14:0	14:3OH:0	226.2
d15:0	15:2OH:0	224.2
d15:1	15:2OH:1	222.2
d15:2	15:2OH:2	220.2
t15:0	15:3OH:0	240.2
d16:0	16:2OH:0	238.2

d16:1	16:2OH:1	236.2
d16:2	16:2OH:2	234.2
t16:0	16:3OH:0	254.2
d17:0	17:2OH:0	252.2
d17:1	17:2OH:1	250.2
d17:2	17:2OH:2	248.2
t17:0	17:3OH:0	268.2
d18:0	18:2OH:0	266.2
d18:1	18:2OH:1	264.2
d18:2	18:2OH:2	262.2
t18:0	18:3OH:0	282.2
d19:0	19:2OH:0	280.2
d19:1	19:2OH:1	278.2
d19:2	19:2OH:2	276.2
t19:0	19:3OH:0	296.2
d20:0	20:2OH:0	294.2
d20:1	20:2OH:1	292.2
d20:2	20:2OH:2	290.2
t20:0	20:3OH:0	310.2

Table S2. List of SP measured in this study across the 12 conditions, including the 7 different species of protozoan parasites, *L. donovani*, the salivarian *Trypanosoma* – *T. vivax*, *T. evansi*, *T. congolense*, *T. brucei* and the stercorarian *Trypanosoma*, *T. rangeli* and *T. cruzi*. For *T. brucei*, two subspecies, *T. brucei rhodesiense* and *T. brucei brucei*, were included. All parasite species were available in mammalian forms, with the exception of *T. rangeli*, which was cultured in the insect form. The lifecycle stage-dependent changes in SP were evaluated with the analyses of insect and mammalian forms of *T. brucei* and *T. cruzi*. All SP species were expressed as mol% of total SP measured.
